# Haplotype analysis of the mitochondrial DNA *d‐loop* region reveals the maternal origin and historical dynamics among the indigenous goat populations in east and west of the Democratic Republic of Congo

**DOI:** 10.1002/ece3.8713

**Published:** 2022-03-14

**Authors:** Patrick Baenyi Simon, Joseph Owino Junga, Getinet Mekuriaw Tarekegn, Eunice Machuka, Christian Keambou Tiambo, Dorine Kabange, Katunga Musale M. Dieudinné, Roger Vumilia Kizungu, Joel Winyo Ochieng, Roger Pelle

**Affiliations:** ^1^ Department of Animal Production Université Evangélique en Afrique Bukavu Democratic Republic of Congo; ^2^ 107854 Department of Animal Production University of Nairobi Nairobi Kenya; ^3^ Institut National d'Etudes et des Recherches Agronomiques INERA Mulungu Bukavu Democratic Republic of Congo; ^4^ Bioscience Eastern and Central Africa‐International Livestock Research Institute (BecA‐ILRI) Hub Nairobi Kenya; ^5^ Department of Animal Breeding and Genetics Swedish University of Agricultural Sciences Uppsala Uppsala Sweden; ^6^ Department of Animal Production and Technology Bahir Dar University Bahir Dar Ethiopia; ^7^ Centre for Tropical Livestock Genetics and Health (CTLGH) – ILRI Nairobi Kenya; ^8^ 121543 Department of Veterinary Medicine Université de Lubumbashi Lubumbashi Democratic Republic of Congo; ^9^ Institut National d’Etudes et des Recherches Agronomiques INERA, Gombe Kinshasa Kinshasa Democratic Republic of Congo

**Keywords:** Democratic Republic of Congo, genetic diversity, haplogroup, mismatch distribution pattern, population expansion

## Abstract

This study aimed at assessing haplotype diversity and population dynamics of three Congolese indigenous goat populations that included Kasai goat (KG), small goat (SG), and dwarf goat (DG) of the Democratic Republic of Congo (DRC). The 1169 bp *d*‐*loop* region of mitochondrial DNA (mtDNA) was sequenced for 339 Congolese indigenous goats. The total length of sequences was used to generate the haplotypes and evaluate their diversities, whereas the hypervariable region (HVI, 453 bp) was analyzed to define the maternal variation and the demographic dynamic. A total of 568 segregating sites that generated 192 haplotypes were observed from the entire *d*‐*loop* region (1169 bp *d*‐*loop*). Phylogenetic analyses using reference haplotypes from the six globally defined goat mtDNA haplogroups showed that all the three Congolese indigenous goat populations studied clustered into the dominant haplogroup A, as revealed by the neighbor‐joining (NJ) tree and median‐joining (MJ) network. Nine haplotypes were shared between the studied goats and goat populations from Pakistan (1 haplotype), Kenya, Ethiopia and Algeria (1 haplotype), Zimbabwe (1 haplotype), Cameroon (3 haplotypes), and Mozambique (3 haplotypes). The population pairwise analysis (*F_ST_*) indicated a weak differentiation between the Congolese indigenous goat populations. Negative and significant (*p*‐value <.05) values for *F*u's *F*s (−20.418) and Tajima's (−2.189) tests showed the expansion in the history of the three Congolese indigenous goat populations. These results suggest a weak differentiation and a single maternal origin for the studied goats. This information will contribute to the improvement of the management strategies and long‐term conservation of indigenous goats in DRC.

## Graphical abstract

The analysis of mtDNA *d*‐*loop* region of three Congolese indigenous goat populations reveals a weak differentiation between them. They were clustered into the haplogroup A with one maternal origin. Demographic expansion was observed in the studied Congolese goat populations.

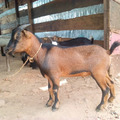

## 1 INTRODUCTION

In most developing countries, agriculture and particularly livestock farming constitute an important source of income in rural households (Herrero et al., 2013). In that respect, goats, one of the first domesticated animals, provide meat and milk as a major source of income for smallholder farmers (Aziz, 2010; Baenyi Simon et al., 2021; Naderi et al., 2008; Skapetas & Bampidis, 2016).

Estimated to 4,065,709 heads, indigenous goat populations in DRC are grouped into three local breeds locally called “chèvre *moyenne du Congo*” *or* small goat (SG) of Congo, “*chèvre du Kasai*” or Kasai goat (KG), and “*chèvre de Bandundu*” or dwarf goat (DG) of Congo (FAOSTAT, 2015; Lafleur et al., 2018; accessed March, 2021). These goats are spread throughout all the agro‐ecological zones (AEZs) of the country where they are kept by farmers (FAOSTAT, 2015; accessed March 2021). In South Kivu, goats contribute up to 40% to farmers’ household income (Baenyi Simon et al., 2021; Wasso et al., 2018). As in eastern Africa, goats in DRC are raised in marginal areas, where crops production is not possible, in different production systems with the predominance of an extensive system that is characterized by low breeding inputs (Muigai et al., 2017).

Due to uncontrolled livestock movements across borders, exotic goat breeds would have been introduced and crossbred with the three Congolese indigenous goat populations. Such a practice increases the risk of the disappearance of resilient and adapted local breeds. To mitigate this risk, the locally adapted goat breeds in DRC need to be characterized, conserved, and utilized sustainably. Characterization of local breeds provides large knowledge and gives a clear perspective on the population structure that will assist in the decision‐making of future breeding programs (Groeneveld et al., 2010; Yang et al., 2016). Because animal mitochondrial DNA (mtDNA) evolves faster than a nuclear genetic marker, it represents a good informative region for the study of phylogenetic and evolutionary biology (Ladoukakis & Zouros, 2017). It also permits the faster examination of the relatedness of populations and has become important in biogeographic and anthropologic studies (Lehman & Fleagle, 2006). The mtDNA polymorphism, especially the displacement loop (*d*‐*loop*) region, is one of the important tools that have been used to better understand the genetic diversity, the population structure, and the population dynamics in different animal species including goats (Phyu et al., 2017; Tarekegn et al., 2018, 2019), sheep (Agaviezor et al., 2012), and chickens (Liu et al., 2009).

In general, six mtDNA haplogroups (A, B (B1 and B2), C, D, F, and G) were identified and found distributed in different geographic areas in the world. The haplogroup A was shown to have a large geographic distribution (Pereira et al., 2005) and was more reported in a large part of African regions (Luikart et al., 2001). However, haplogroups B and G were also reported in some African countries; with haplogroup B particularly found limited in the South part of Africa, especially in South Africa and Namibia, while the haplogroup G was reported in Egypt (Naderi et al., 2007), Kenya (Kibegwa et al., 2016), Ethiopia (Tarekegn et al., 2018), Sudan, and Somalia (Al‐Araimi et al., 2017). A previous study on indigenous goat from *Peste des Petits Ruminants* outbreak zones in South Kivu province of DRC based on mtDNA *d*‐*loop* variation revealed the presence of two haplogroups A (commonest) and B within the goat population in South Kivu (Bwihangane et al., 2018). However, the result from this study was limited to goats from *Peste des Petits Ruminants* outbreak zones in the South Kivu region and could not reveal more information on the genetic diversity of indigenous goat breeds in the whole country and did not mention the goat populations’ dynamics and history. Therefore, this study aimed to describe the haplotype diversity, the population structure, and the demographic dynamics of three indigenous goat breeds in three AEZs of DRC based on the mtDNA *d*‐*loop* region.

## 2 MATERIALS AND METHODS

### 2.1 Sampling and DNA extraction

**FIGURE 1:**
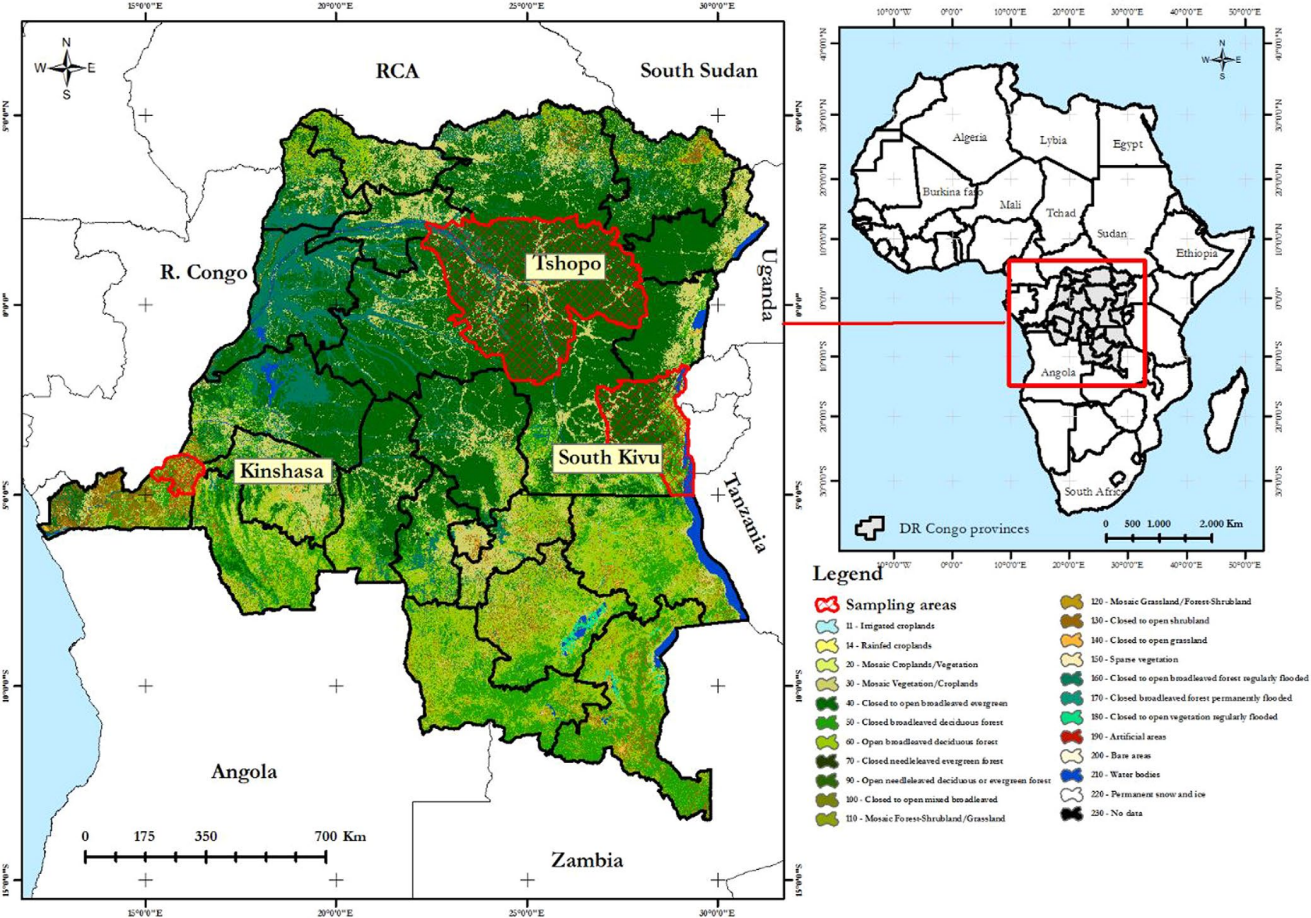
Sampling areas in the Democratic Republic of Congo

Sampling was conducted in collaboration with the Ministry of Agricultural, Livestock, and Fisheries of DRC through the representative inspections in each sampling region that included Kinshasa, Tshopo, and South Kivu (Figure 1).

A total of 339 blood samples representing three Congolese indigenous goat populations (Kasai goat, *n* = 108; dwarf goat, *n* = 114 and small goat, *n* = 117) were sampled from farmer's flocks from the three AEZs of DRC (representing in this study by Kinshasa, Tshopo and South Kivu) and used for the study. Efforts were made to avoid closely related individuals during sampling. The socioeconomic factors associated with goat keeping (Wasso et al., 2018), the environmental characteristics (the high land volcanic mountain), and the proximity to neighboring countries (Tanzania, Rwanda, and Burundi, with which animal exchanges can readily occur leading to an uncontrolled inter crossbreeding between goat populations) were the main reasons for choosing the South Kivu region. Tshopo was chosen based on its geographic location (the equatorial forest region) which could affect goat management and productivity, while Kinshasa (capital city) was chosen based on the environmental characteristic (high temperature), the commercial transaction with surrounding regions including Bandundu, Kasai central, and Congo central, and the productivity history of goats (Gasigwa Sabimana et al., 2017).

Genomic DNA was extracted from blood samples using the QIAamp^®^ DNA Mini kit (Qiagen) according to the manufacturer's protocol. DNA quality control (QC) was done using a spectrophotometer (NanoDrop 2000; Thermo Fisher Scientific, USA) and DNA integrity was checked on 1% agarose gel electrophoresis.

### 2.2 PCR amplification and sequencing of the *d*‐*loop* region

The 1169 bp of the mtDNA *d*‐*loop* region was amplified using primers designed (F: 5’‐ACCAGAAAAGGAGAATAGCC‐3’; R: 5’–GGTACACTCATCTAGGCATT‐3’) using a three‐step PCR. PCRs were carried out in 25 µl reaction volumes composed of Phusion master mix (2× concentrated solution which included Taq DNA polymerase (0.05 U/µl), reaction buffer, 4 mM MgCl_2_, and 0.4 mM of each dNTP), 0.2 µM of each primer (F and R), 2% of dimethyl sulfoxide (DMSO), and 40 ng of template DNA. The three‐step PCR involved an initial denaturation at 98°C for 30 s followed by 35 cycles of amplification (denaturation at 98°C for 10 s, annealing at 61°C for 30 s, and extension at 72°C for 30 s) and completed by the final extension step at 72°C for 7 min. The PCR products were purified using the QIAquick^®^ PCR purification kit (Qiagen) following the manufacturer's protocol. The reverse primer (R: 5’‐GGTACACTCATCTAGGCATT‐3’) and a pair of GDLS2 primers (GDLS‐2F: 5’‐ACCTAAAATCGCCCACTC‐3’; GDLS‐2 R: 5’‐TGATCTAGTGGACGGGATAC‐3) were respectively used as external and internal primers to sequence the purified PCR products (Tarekegn et al., 2018).

### 2.3 Data analysis

Default values and parameters inherent in algorithms and software were used for all analyses undertaken in this study. Only deviations from the default were mentioned. Before the analyses, all the chromatograms were visualized using CLC Genomics workbench v8.0 software. MEGA v6.4 software was used for the multiple sequence alignments with the ClustalW algorithm (Tamura et al., 2013). The variable sites were scored against the *Capra hircus* reference sequence (GenBank accession number: GU223571: direct submission). In total, 339 sequences were generated from where haplotypes were generated with DnaSP v5 (Rozas et al., 2003). Genetic diversity parameters that include the number of haplotypes (*N*), haplotype diversity (*H_d_*), nucleotide diversity (*π*), and mean of nucleotide differences between haplotypes (*K*) and their standard deviations (SD) were analyzed for each goat population and across all populations using DnaSP v5 (Librado & Rozas, 2009; Rozas et al., 2003).

A phylogenetic tree was constructed using haplotypes generated in Congolese indigenous goat and 22 reference haplotypes representing the 6 haplogroups (A, B, C, D, G, and F) defined based on the variation in the first HVI with 481 bp of length size (Luikart et al., 2001; Naderi et al., 2007, 2008) corresponding to positions 15,709–16,190 bp of the *Capra hircus* mtDNA reference sequence (Gene Bank accession number GU295658) with the neighbor‐joining (NJ) algorithm implemented in MEGA v6.4 with the level of confidence associated with each bifurcation evaluated with 1000 bootstrap replications. To visualize Congolese indigenous goats in the context of the regional and global caprine diversity and obtain further insights into genetic relationships between the haplotypes, a total of 336 published sequences and 22 reference sequences of domestic goats representing the 6 globally defined mtDNA *d*‐*loop* haplogroups (Naderi et al., 2007) were retrieved from the GenBank (Table S1) and included in the NJ tree and MJ network analyses using Network v4.6 software (Bandelt et al., 1999). The added sequences were from 13 African countries: Cameroon (central Africa), Kenya, Ethiopia (East Africa), Egypt, Algeria, Libya, Tunisia and Morocco (North Africa), Senegal, Nigeria (West Africa), Namibia, Zimbabwe, and Mozambique (southern Africa). In addition, sequences from 10 Asian countries (India, Iraq, Saudi Arabia, Pakistan, China, Laos, Iran, Mongolia, Jordan, and Azerbaijan) and 6 European countries (Turkey, Austria, France, Italy, Switzerland, and Spain) were included in the analysis.

The genetic variation among Congolese indigenous goat populations was evaluated through the analysis of molecular variance (AMOVA) following 1000 permutations in Arlequin v3.5.2 (Excoffier & Lischer, 2010). Pairwise genetic differentiations (*F_ST_*) (Reynolds et al., 1983) were estimated between each of the three Congolese indigenous population and a group consisting of the non‐Congolese goat populations grouped according to the geographical regions using Arlequin v3.5.2 software (Excoffier & Lischer, 2010) with the number of permutations for significance estimated at 100 at the significance level of 0.05.

We inferred population demographic history and dynamics from haplotype mismatch distribution patterns (Rogers & Harpending, 1992) and the expected distributions plus their 95% confidence intervals for the three Congolese indigenous goat populations. Departures of the observed sum of squares differences (SSD) from the simulated model of expansion were tested with the chi‐square test of goodness of fit statistic and Harpending's raggedness index “*r*” (Harpending, 1994) following 1000 coalescent simulations. Analysis of mismatch distribution patterns was augmented with two coalescent‐based estimators of neutrality: Fu's *F*s (Fu, 1997) and Tajima's *D* (Tajima, 1989) statistics. The significance of these two statistics was tested with 1000 coalescent simulations in Arlequin v3.5.2.

## 3 RESULTS

### 3.1 mtDNA *d*‐*loop* sequence variation and genetic diversity

From the 339 generated sequences of the mtDNA *d*‐*loop* region representing the three Congolese indigenous goat populations spanning the entire 1169 bp, a total of 192 haplotypes defined by 568 segregating sites were detected from the *d*‐*loop* region of these sequences aligned against the caprine reference (Accession number GU223571). Out of the 192 haplotypes, 23 were shared between the Congolese indigenous goat populations (seven haplotypes shared between the three Congolese indigenous goat populations, eight shared between Kasai goat and small goat, three between Kasai goat and dwarf goat, and five between dwarf goat and small goat). Furthermore, nine haplotypes were shared between the three Congolese indigenous goat populations and the goat populations in Africa. These include one haplotype (mostly represented by a small goat) shared with goat populations from Ethiopia, Kenya, and Algeria; three haplotypes mostly represented by dwarf goat shared with goat populations from Cameroon; and three and one haplotypes mostly represented by Kasai goat were respectively shared with goat populations from Mozambique and Zimbabwe. Outside Africa, one haplotype was shared with Pakistan goat.

The three Congolese indigenous goat populations showed high levels of genetic diversity (average haplotype diversity: 0.994 ± 0.03 for small goat, 0.994 ± 0.003 for Kasai goat, and 0.973 ± 0.007 for dwarf goat). The average nucleotide diversity was higher for dwarf goat (*π* = 0.018 ± 0.004) than for small goat (*π* = 0.013 ± 0.002) and Kasai goat (*π* = 0.012 ± 0.022) (Table 1).

**TABLE 1:** mtDNA *d*‐*loop* sequence variation and genetic diversity for Congolese goats

Population	*N*	*S*	*H*	*H_d_* ± SD	*π* ± SD	*K*
Kasai goat	108	187	91	0.994 ± 0.003	0.012 ± 0.022	13.899
Small goat	117	200	96	0.994 ± 0.03	0.013 ±0.002	14.806
Dwarf goat	114	495	60	0.973 ± 0.007	0.018 ± 0.004	18.104
All population	339	568	192	0.987 ± 0.002	0.015 ± 0.003	14.740

Abbreviations: *N*, Number of samples per populations; *S*, number of segregating sites; *H*, number of haplotypes detected; *H_d_*, haplotype diversity; SD, standard deviation; *π*, nucleotide diversity; *K*, average number of nucleotide differences; KG, Kasai goat (Kinshasa); SG, small goat (South Kivu); DG, dwarf goat (Tshopo).

### 3.2 Population phylogenetic and relationship analysis

**FIGURE 2:**
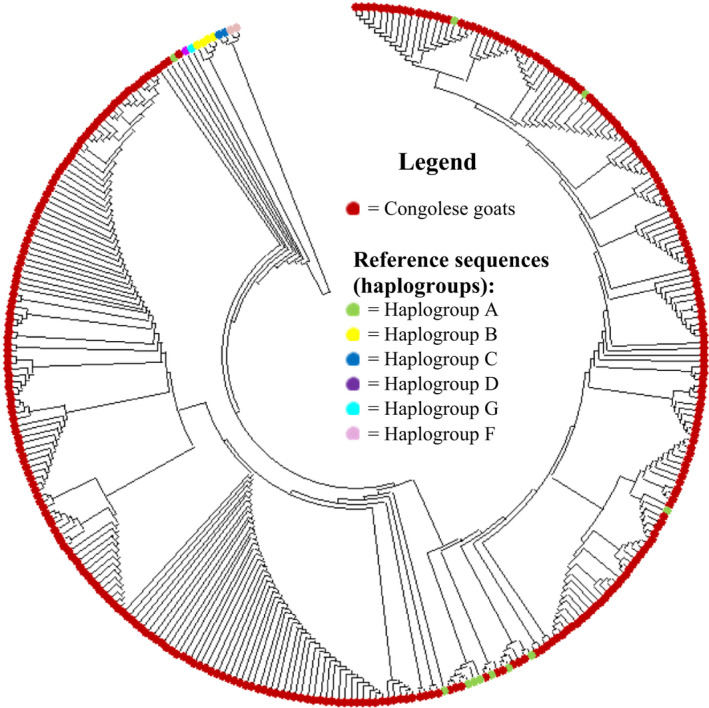
Neighbor‐joining tree constructed using the HVI region of the mtDNA *d*‐*loop* of three Congolese indigenous goat populations including reference haplotypes representing six globally defined haplogroups (a, b, c, d, g, and f) observed in goats (Naderi et al., 2007). The tree was inferred from aligned nucleotide sequences by the neighbor‐joining method at Bootstrap 1000 replicates implemented in the MEGA v6.4 software

**FIGURE 3:**
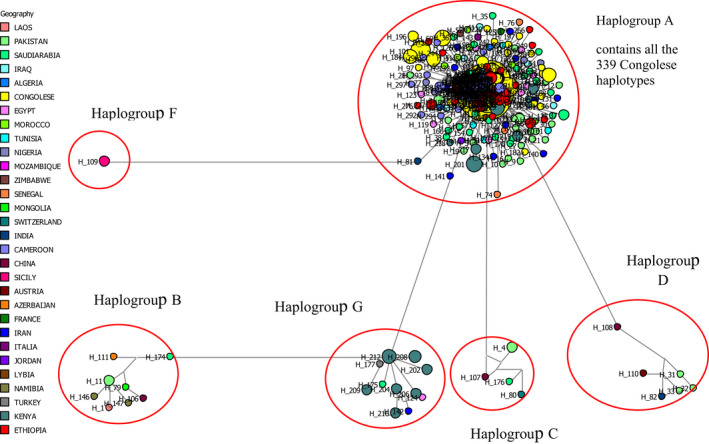
Median‐joining network analyses based on HIV region of 339 haplotypes of Congolese indigenous goat populations and 336 haplotypes of reference sequences from 29 different countries (13 African countries, 10 Asian countries, and 6 European countries) and which represent the 6 predefined haplogroups

The first hypervariable (HVI, 453bp) sequences both for Congolese indigenous goat populations and the reference sequences representing the six domestic goat haplogroups were aligned using the ClustalW algorithm implemented in MEGA v6.4 and considered for the NJ tree analysis. All the three Congolese indigenous goat populations (100%) were clustered into mtDNA lineage A (Figure 2). An MJ (as described in the data analysis section) constructed to provide a wider resolution of the phylogenetic relationship between the Congolese indigenous goat populations and the non‐Congolese goats (Figure 3) supported the result obtained by the NJ tree analysis.

### 3.3 Population genetic structure

The AMOVA for the three Congolese indigenous goat populations grouped by AEZs revealed that 5.88% of the total genetic variation was attributed to the genetic differences among populations and the highest proportion (94.12%) to variation within populations (Table 2). The pairwise genetic distance (F*_ST_*) value revealed low genetic differentiation between the three studied Congolese indigenous goat populations with *F*_ST_ = 0.02165; 0.09182; 0.04567 respectively observed between Kasai and dwarf goat, Kasai and small goat, and small and dwarf goat (Table 3). In the context of the regional and global caprine diversity, the *F*_ST_ values observed between Congolese goat populations were lower compared to these observed between the Congolese and non‐Congolese goat populations with *F*_ST_ = 0.23052; 0.66525; 0.20138; 0.23111; 0.21406; 0.20800; 0.35904 respectively observed between Congolese and Asian, European, Northern African, Eastern African, Western African, Cameroonian, and Southern African goat populations (Table 4).

**TABLE 2:** Analysis of molecular variance (AMOVA)

Source of variation	Degrees of freedom	Sum of squares	Variance components	Percentage of variation
Among populations	2	75.740	0.44749	5.88
Within population	337	2415.254	7.16693	94.12
Total	339	2490.994	7.61442	

**TABLE 3:** Population pairwise (*F*_ST_) to estimate the genetic distance between the three Congolese goat populations

Population	Kasai goat (KG)	Dwarf goat (DG)	Small goat (SG)
Kasai goat (KG)	0.00000		
Dwarf goat (DG)	0.02165	0.00000	
Small goat (SG)	0.09182	0.04567	0.00000

**TABLE 4:** Population pairwise (*F*_ST_) to estimate the genetic distance between the Congolese and the non‐ Congolese goat populations

Goat populations	Asia	Europe	North Africa	East Africa	West Africa	Cameroon	Southern Africa	DRC
Asia	0.00000							
Europe	0.23761	0.00000						
North Africa	0.01467	0.32033	0.00000					
East Africa	0.03722	0.33168	0.02473	0.00000				
West Africa	0.00902	0.26289	−0.01361	0.02910	0.00000			
Cameroon	0.16821	0.62990	0.21757	0.18638	0.19294	0.00000		
Southern Africa	0.10085	0.20189	0.15630	0.15075	0.13509	0.30033	0.00000	
DRC	0.23052	0.66525	0.20138	0.23111	0.21406	0.20800	0.35904	0.00000

### 3.4 Population dynamics and history

**FIGURE 4:**
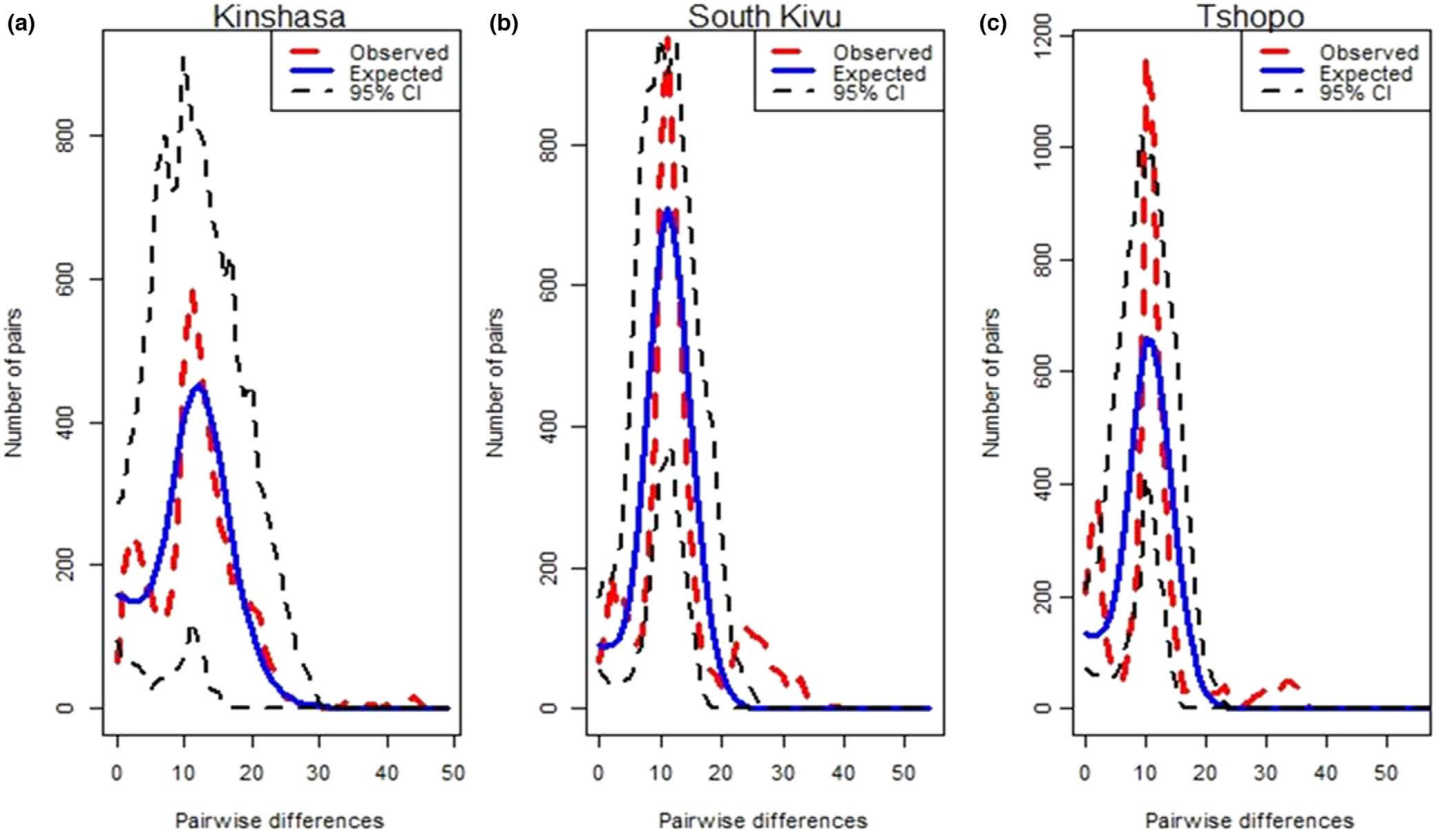
Demographic dynamics among the indigenous goat populations in DRC. The demographic dynamic of each population was inferred from mismatch distribution patterns following 1000 coalescent simulations and their 95% confidence interval. (a) Kinshasa (Kasai goat), (b) South Kivu (small goat) and Tshopo (dwarf goat)

Mismatch distribution patterns were used to assess the population dynamics of the three Congolese indigenous goat populations grouped into the haplogroup A as revealed by the NJ tree and the MJ network. For each population, the expansion modal of mismatch distribution was bimodal and a valid goodness of fit was observed between observed and expected distributions plus their 95% confidence intervals, indicating a demographic expansion signal (Figure 4). For the global dataset incorporating the three Congolese indigenous goat populations, the observed patterns of mismatches did not deviate significantly from that expected under a null hypothesis either with the demographic (*p*‐value >.05) or spatial (*p*‐value >.05) expansion modal (Table 5). Exception was observed for the dwarf and small goats for which the SSD values were significant (*p*‐value <.05). The variations around the curves were also not significant except for dwarf goats (*p*‐value <.01) if considering the demographic expansion modal (Table 5). However, the negative Tajima's *D* (−2.189) and Fu's *F*s (−20.418) and significant (*p*‐value <.05) values obtained suggest that the three Congolese indigenous goats have expanded demographically in their past.

**TABLE 5:** Population demographic parameters estimated from the mismatch analysis of the mtDNA *d*‐*loop* in 3 Congolese goat populations

Population	*N*	*S*	Demographic expansion		Spatial expansion		Tajima's D (*p*‐value)	Fus’ *F*s (*p*‐value)
			SSD (*p*‐value)	Raggedness index (*p*‐value)	SSD (*p*‐value)	Raggedness index (*p*‐value)		
Kasai goat	108	187	0.003 (0.510)	0.003 (0.820)	0.007 (0.200)	0.003 (0.850)	−1.980 (0.000)	−24.262 (0.000)
Dwarf goat	114	495	0.023 (0.000)	0.022 (0.00)	0.249 (0.000)	0.022 (0.080)	−2.705 (0.000)	−12.818 (0.021)
Small goat	117	200	0.009 (0.010)	0.008 (0.070)	0.010 (0.000)	0.008 (0.240)	−1.881 (0.014)	−24.173 (0.000)
All	339	568	0.012 (0.173)	0.011 (0.296)	0.014 (0.066)	0.011 (0.390)	−2.189 (0.046*)	−20.418 (0.007*)

Abbreviations: *N*, Sample sizes; *S*, segregating sites; SSD, sum of squared deviations.

*Statistically significant (*p* < .05).

## 4 DISCUSSION

Here, sequence variations of the mtDNA *d*‐*loop* region of three Congolese indigenous goat populations from eastern and western DRC were analyzed to assess the genetic diversity, the genetic differentiation, and the demographic historical profiles. The results revealed 192 haplotypes from the analyses of 339 mtDNA *d*‐*loop* sequences. Average haplotype diversity and nucleotide diversity of 0.987 ± 0.002 and 0.015 ± 0.003 respectively were obtained for the three populations with an average number of nucleotide differences of 14.740. This average haplotype diversity is similar to that of Kenyan goats (0.981; Kibegwa et al., 2016), lower than that of Cameroonian (0.995 ± 0.002; Tarekegn et al., 2019), Ethiopian (0.997 ± 0.001; Tarekegn et al., 2018), European (0.994), and Iberian (0.996) goats but higher than that of South and Central American (0.963; Amills et al., 2009) and Sicilian (ranged between 0.86 and 0.969; Sardina et al., 2006) goats. These results assume that the maternal genetic diversity of Congolese indigenous goats is not at risk of loss neither through extinction nor through genetic erosion. This assumes that they are potential resources to use in the designing of goat conservation and improvement programs in the Country. The highest level of haplotype diversity and segregating sites in small goat (South Kivu) compared with the two other populations (Kasai goat and dwarf goat) suggests its adaptation ability in wide‐range environments of DRC.

Out of 192 haplotypes observed in this study, 7 haplotypes were common to all the 3 Congolese indigenous goat populations with 1 haplotype having the highest frequency (42 individuals). This result may be attributed to the evolutionary relationship among the studied goat populations. Relatively divergent haplotypes within breeds and geographical locations suggest that the gene flow has occurred on a regional scale during some time in the recent past and the breeds have not been subdivided by long‐term biogeographic barriers (Luikart & Allendorf, 1996). In a study conducted by Vacca et al. (2010), it has been reported that goats have shown high genetic haplotype diversity, from where more haplotypes were each represented by a single sequence and only a few were shared among animals. Results in this study support that finding and the findings of Naderi et al. (2007) showing that it is common to find haplotypes represented by one individual or shared by only a few individuals. Based on the available goat mitochondrial haplogroup classification system (Naderi et al., 2007) and by incorporating reference haplotypes, only haplogroup A was found among the three studied Congolese indigenous goat populations. Therefore, Bwihangane et al. (2018) have found one individual from the Fizi goat population (0.9%) to be aligned to lineage B in the total individuals considered (110; 100%) in the study. Since Fizi is closed to Tanzania (eastern country) from where the haplogroup B was found in goat populations (Nguluma et al., 2021), the probability of having the goat aligned to the lineage B could be possible. Further investigation is required to confirm the presence of haplogroup B in Congolese indigenous goat populations in that area of South Kivu. The haplogroup A has been shown to have a large geographic distribution (Pereira et al., 2005) and was more reported in large parts of African regions (Luikart et al., 2001). That result could be interpreted as evidence that Congolese indigenous goats come from a unique maternal population with one maternal lineage which could have been introduced from one geographic domestication area (Naderi et al., 2008). On the one hand, the fact that 3 and 1 haplotypes were shared between Congolese and Mozambican, and Congolese and Zambian goats, respectively, suggest that Congolese goats might have been descended from southern African goat populations. On the other hand, based on the number of haplotypes shared between Congolese, Cameroonian, Ethiopian, and Kenyan goats, we may deduce that these goats might have the same origin or the Congolese indigenous goats might have been descended either from western or eastern Africa. Based on the number of haplotypes (3) (in this study) shared with Congolese indigenous goats and based on the genetic differentiation (F*_ST_*, 0.20800) observed between Congolese and Cameroonian goats (Table 4), we may consider that Congolese and Cameroonian (central African countries) goats are genetically closed and may have descended from the same origin occurred in closed periods.

The AMOVA showed that 5.88% represented the genetic variation among populations in Congolese indigenous goat populations compared to 94.12% within populations. The higher within‐population variation than among populations could be associated with the breeding practices. In addition, it can suggest high levels of female‐mediated gene flow (Moritz et al., 1987; Tserenbataa et al., 2004) and relates to high haplotype diversity implying widespread distribution and diversity to favor for selection within populations (Kibegwa et al., 2016). A weak phylogeography with small genetic differentiation (*F*_ST_) was confirmed between the three studied Congolese indigenous goat populations (Table 3). This low genetic differentiation points to a high historical gene flow and intermixing between the three goat populations studied. Consistency is observed between the result in this study and previous reports confirming that weak genetic structure is most observed in small ruminants (sheep and goat) than in large ruminants (cattle) (Luikart et al., 2001). Thus, findings in this study suggest that the three Congolese indigenous goat populations share a relatively similar genetic background with the same maternal origin as revealed by the demographic dynamics inferred from the mismatch distribution pattern (Figure 4). The mismatch distribution patterns were bimodal for the three Congolese indigenous goat populations. Similar demographic patterns have been observed in Tanzanian (Nguluma et al., 2021), Ethiopian (Tarekegn et al., 2018), and Oman indigenous goats (Al‐Araimi et al., 2017). The negative and significant (*p*‐value <.05) Tajima's *D* (−2.189) and Fu's *D* (−20.418) values supported population expansion for all the three Congolese indigenous goat populations. These results suggest that the studied goat populations underwent an expansion. The significant departure (*p*‐value <.05) observed for the Tajima's *D* and Fu's *F*s for all populations explained mainly an excess of new mutations corresponding to the results of evolutionary forces due to either selective sweeps or population growth.

## 5 CONCLUSION

This is the first study that investigates the genetic diversity within and between the three Congolese indigenous goat populations from east and west of the Democratic Republic of Congo. The analyses of the mtDNA control region (*d*‐*loop*) revealed a high level of genetic diversity in the east and west Congolese indigenous goats with a weak genetic differentiation, and a unique maternal origin belonging to haplogroup A. The three indigenous goat populations share a relatively similar genetic background. The demographic expansion was observed in the studied Congolese goat populations. These results represent an abundant resource for selective breeding in the different AEZs of the DRC.

## CONFLICT OF INTEREST

No conflict of interest was reported by the author(s).

## AUTHOR CONTRIBUTIONS

**Patrick Baenyi Simon:** Conceptualization (equal); Data curation (equal); Formal analysis (lead); Investigation (lead); Methodology (lead); Software (equal); Writing – original draft (equal); Writing – review & editing (equal). **Joseph Owino Jungá:** Conceptualization (equal); Formal analysis (supporting); Investigation (supporting); Project administration (equal); Supervision (lead); Validation (equal); Writing – review & editing (equal). **Getinet Mekuriaw Tarekegn:** Conceptualization (equal); Formal analysis (supporting); Methodology (equal); Software (equal); Supervision (equal); Validation (equal); Visualization (equal); Writing – review & editing (equal). **Eunice Machuka:** Data curation (equal); Methodology (equal); Validation (equal); Writing – review & editing (equal). **Christian Keambou Tiambo:** Investigation (supporting); Methodology (supporting); Supervision (equal); Validation (equal); Writing – review & editing (equal). **Dorine Kabange:** Data curation (supporting); Methodology (supporting); Writing – review & editing (equal). **Katunga Musale M. Dieudinné:** Investigation (supporting); Writing – review & editing (equal). **Roger Vumilia Kizungu:** Investigation (supporting); Validation (equal); Writing – review & editing (equal). **Joel Winyo Ochieng:** Formal analysis (supporting); Investigation (supporting); Methodology (supporting); Supervision (equal); Validation (equal); Writing – review & editing (equal). **Roger Pelle:** Conceptualization (equal); Data curation (supporting); Formal analysis (supporting); Funding acquisition (lead); Investigation (supporting); Methodology (supporting); Project administration (lead); Resources (lead); Supervision (lead); Validation (equal); Writing – review & editing (equal).

## Supporting information

Table S1

## Data Availability

Mitochondrial sequence data generated as part of this project are deposited in dryad and given accession number https://doi.org/10.5061/dryad.vq83bk3v6. Other mtDNA sequences incorporated into the analysis were downloaded from this source and can be retrieved as per the relevant citations.
