# Supplementary material for: Haplotype analysis of the mitochondrial DNA d‐loop region reveals the maternal origin and historical dynamics among the indigenous goat populations in east and west of the Democratic Republic of Congo
Source: Ecol Evol. 2022 Mar 14;12(3):e8713. doi: 10.1002/ece3.8713 (PMC8928878; doi:10.1002/ece3.8713)
Supplement: Supplementary file 1 — Table S1 [file ECE3-12-e8713-s001.docx]

| Country of origin | Sample size | Haplogroup | Accession number | Reference |
| --- | --- | --- | --- | --- |
| Laos | 1 | B | AB044303.1 | Mannen et al 2001 |
| Pakistan | 36 | A, B, C, D | AB110552, AB110553, AB110555, AB110557, AB110558, AB110559, AB110560, AB110561, AB110562, AB110563, AB110564, AB110565, AB110566, AB110567, AB110568, AB110569, AB110570, AB110571, AB110572, AB110573, AB110574, AB110575, AB110575, AB110577, AB110578, AB110579, AB110580, AB110581, AB110582, AB110583, AB110584, AB110585, AB110586, AB110587, AB110588, AB110589 | Sultana et al., 2003 |
| Saudi Arabia | 39 |  | AJ317752, AJ317753, AJ317754, AJ317755, AJ317757, AJ317758, AJ317759, EF618309, EF618310, EF618311, EF618312, EF618313, EF618314, EF618315, EF618312, EF618313, EF618314, EF618315, EF618316, EF618314, EF618318, EF618319, EF618320, EF618321, EF618323, EF618324, EF618325, EF618327, EF618328, EF618330, EF618331, EF618332, EF618333, EF618334, EF618337, EF618338, EF618339, EF618341, EF618345, | Luikart et al., 2001 ; Naderi et al., 2007 |
| Iraq | 6 | A | AJ317763, AJ317764, AJ317765, AJ317766, AJ317767, AJ317768 | Luikart et al., 2001 |
| Algeria |  |  | AJ317777, AJ317778, AJ317779 |  |
| Egypt | 18 | A, G | AJ317780, AJ317781, AJ317783, AJ317795, AJ317796, EF617711, EF617712, EF617713, EF617714, EF617715, EF617716, EF617717, EF617719, EF617720, EF617721, EF617723, EF617724, EF617727 | Luikart et al., 2001 ; Naderi et al., 2007 |
| Morocco | 5 |  | AJ317784, AJ317785, AJ317786, AJ317787, AJ317788 |  |
| Tunisia |  |  | AJ317789, AJ317790, AJ317791, AJ317792, AJ317793 |  |
| Nigeria | 4 | A | AJ317801, AJ317810, AJ317823, AJ317825 | Luikart et al., 2001 ; Naderi et al., 2007 |
| Zimbabwe | 2 |  | AJ317802, AJ317803 |  |
| Mozambique | 6 |  | AJ317804, AJ317805, AJ317806, AJ317807, AJ317808, AJ317809 |  |
| Senegal |  |  | AJ317816, AJ317817, AJ317818, |  |
| Mongolia | 1 | B | AJ317833 | Luikart et al 2001 |
| Switzeland | 1 | C | AJ317838.1 | Luikart et al 2001 |
| India | 2 | A, D | AY155721.1, AY155952.1 | Joshi et al., 2004 |
| China | 3 | B, C, D | DQ121578, DQ188892, DQ188893 | Liu et al., 2005 and 2006 |
| Sicily | 2 | F | DQ241349.1, DQ241351.1 | Sardina et al 2006 |
| Austria | 1 | D | EF617701 | Naderi et al 2007 |
| Azerbaijan | 1 | B | EF617706.1 | Naderi et al 2007 |
| France | 1 | A | EF617779.1 | Naderi et al 2007 |
| Iran | 17 | A, G | EF617863, EF617864, EF617865, EF617868, EF617869, EF617870, EF617871, EF617872, EF617873, EF617875, EF617876, EF617878, EF617879, EF617880, EF617945, EF618083, EF618084, | Naderi et al 2007 |
| Italy | 1 | A | EF618134 | Naderi et al 2007 |
| Jordan | 1 | A | EF618200 | Naderi et al 2007 |
| Lybia |  |  | EF618220 |  |
| Namibia |  |  | EF618244, EF618245 |  |
| Spain | 1 | C | EF618413 | Naderi et al 2007 |
| Turkey | 1 | G | EF618535 | Naderi et al 2007 |
| Kenya | 55 | A, G | KP120622, KP120623, KP120624, KP120625, KP120626, KP120627, KP120628, KP120629, KP120629, KP120630, KP120632, KP120634, KP120635, KP120637, KP120638, KP120639, KP120640, KP120641, KP120642, KP120643, KP120646, KP120648, KP120649, KP120650, KP120652, KP120653, KP120654, KP120655, KP120646, KP120648, KP120649, KP120650, KP120652, KP120653, KP120654, KP120655, KP120656, KP120657, KP120658, KP120660, KP120661, KP120666, KP120667, KP120668, KP120669, KP120670, KP120672, KP120673, KP120674, KP120675, KP120677, KP120678, KP120679, KP120680, KP120681 | Kibegwa et al., 2015 |
| Ethiopia | 75 | A, G | KY747687, KY747688, KY747689, KY747690, KY747691, KY747692, KY747693, KY747694, KY747695, KY747696, KY747697, KY747698, KY747699, KY747700, KY747701, KY747702, KY747703, KY747704, KY747705, KY747706, KY747707, KY747708, KY747709, KY747710, KY747711, KY747712, KY747713, KY747714, KY747715, KY747716, KY747708, KY747709, KY747710, KY747711, KY747712, KY747713, KY747714, KY747715, KY747716, KY747717, KY747718, KY747719, KY747720, KY747721, KY747722, KY747723, KY747724, KY747725, KY747727, KY747730, KY747734, KY747738, KY747739, KY747741, KY747743, KY747744, KY747752, KY747756, KY747730, KY747734, KY747738, KY747739, KY747741, KY747743, KY747744, KY747752, KY747756, KY747764, KY747772, KY747773, KY747774, KY747775, KY747778, KY747787, KY747794 | Tarekegn et al., 2018 |
| Cameroon | 53 | A | MH621412, MH621415, MH621418, MH621419, MH621420, MH621421, MH621422, MH621424, MH621425, MH621426, MH621427, MH621428, MH621430, MH621432, MH621433, MH621434, MH621437, MH621438, MH621439, MH621440, MH621441, MH621443, MH621445, MH621446, MH621447, MH621449, MH621451, MH621452, MH621455, MH621456, MH621459, MH621460, MH621463, MH621466, MH621467, MH621468, MH621469, MH621470, MH621471, MH621473, MH621474, MH621477, MH621478, MH621480, MH621483, MH621485, MH621488, MH621490, MH621494, MH621496, MH621497, MH621498, MH621501, | Tarekegn et al., 2018 |
